# Normal forms for Poisson maps and symplectic groupoids around Poisson transversals

**DOI:** 10.1007/s11005-017-1007-2

**Published:** 2017-10-04

**Authors:** Pedro Frejlich, Ioan Mărcuț

**Affiliations:** 1 Universidade Federal do Rio Grande do Sul, Campus Litoral Norte Rodovia RS 030, 11.700 - Km 92, Emboaba, Tramandaí, RS CEP 95590-000 Brazil; 2 IMAPP, Radboud University Nijmegen, 6500 GL Nijmegen, The Netherlands

**Keywords:** Symplectic manifolds, general, Poisson manifolds, Poisson groupoids and algebroids, Lie algebras and Lie superalgebras, 53D05, 53D17, 17Bxx

## Abstract

Poisson transversals are submanifolds in a Poisson manifold which intersect all symplectic leaves transversally and symplectically. In this communication, we prove a normal form theorem for Poisson maps around Poisson transversals. A Poisson map pulls a Poisson transversal back to a Poisson transversal, and our first main result states that simultaneous normal forms exist around such transversals, for which the Poisson map becomes transversally linear, and intertwines the normal form data of the transversals. Our second result concerns symplectic integrations. We prove that a neighborhood of a Poisson transversal is integrable exactly when the Poisson transversal itself is integrable, and in that case we prove a normal form theorem for the symplectic groupoid around its restriction to the Poisson transversal, which puts all structure maps in normal form. We conclude by illustrating our results with examples arising from Lie algebras.

## Introduction

Poisson transversals are special submanifolds which play in Poisson Geometry a role similar to that of symplectic submanifolds in Symplectic Geometry, and complete transversals in Foliation Theory. A Poisson transversal of a Poisson manifold $(M,\pi )$ is an embedded submanifold $X\subset M$ which intersects all symplectic leaves transversally and symplectically. These submanifolds lie at the heart of Poisson geometry, appearing in many constructions and arguments already since the foundational work of Alan Weinstein [22].

In our previous note [10], we described a normal form theorem around a Poisson transversal $(X,\pi _X)$ in $(M,\pi )$, which depends only on the restriction of $\pi$ to $T^{*}M|_X$. Choosing a Poisson spray $\mathcal {V}$ for $\pi$, the corresponding exponential map induces the Poisson isomorphism around *X* which puts the structure in normal form:

1 $$\begin{aligned} \exp _{\mathcal {V}}:(N^{*}X,\pi _X^{\omega _{\mathcal {V}}}) \hookrightarrow (M,\pi ). \end{aligned}$$

Here $\pi _X^{\omega _{\mathcal {V}}}$ stands for the Poisson structure corresponding to the Dirac structure $p^*(L_{\pi _X})^{\omega _{\mathcal {V}}}$ obtained as follows: by first pulling back the Dirac structure $L_{\pi _X}$ corresponding to $\pi _X$ to $N^*X$ via the map *p*, and then gauge-transforming by a certain closed two-form $\omega _{\mathcal {V}}$ on $N^*X$ which is symplectic on the fibers of *p*. Actually, all these objects ($\exp _{\mathcal {V}}$, $\omega _{\mathcal {V}}$ and $\pi _X^{\omega _{\mathcal {V}}}$) are only defined on a small open neighborhood of $X \subset N^*X$, but we omit this technicality from the notation. The procedure in [10] for constructing normal forms as in (1) depends only on the choice of $\mathcal {V}$, and has the added benefit of allowing simultaneous normal forms for all Poisson transversals in $(M,\pi )$.

In this communication, we continue our analysis of local properties around Poisson transversals with normal form results for Poisson maps and symplectic groupoids.

That Poisson transversals behave functorially with respect to Poisson maps has already been pointed out in [10]: a Poisson map pulls back Poisson transversals to Poisson transversals, and in fact, it pulls back the corresponding infinitesimal data pertaining to their normal forms. We prove that the two Poisson structures and the Poisson map can be put in normal form simultaneously:

### Theorem 1

(Normal form for Poisson maps) Let $\varphi : (M_0,\pi _0) \rightarrow (M_1,\pi _1)$ be a Poisson map, and $X_1 \subset M_1$ be a Poisson transversal. Then $\varphi$ is transverse to $X_1$, $X_0:=\varphi ^{-1}X_1$ is a Poisson transversal in $(M_0,\pi _0)$, $\varphi |_{X_0}:(X_0,\pi _{X_0}) \rightarrow (X_1,\pi _{X_1})$ is a Poisson map, and there exist Poisson sprays $\mathcal {V}_i$ with exponential maps $\mathrm {exp}_{\mathcal {V}_i}:(N^{*}X_i,\pi _{X_i}^{\omega _{\mathcal {V}_i}}) \hookrightarrow (M_i,\pi _i)$ which fit into the commutative diagram of Poisson maps:where *F* is the vector bundle map:

$$\begin{aligned} F_{x}:=(\varphi ^*|_{N^*_{\varphi (x)}X_1})^{-1} : N^{*}_xX_0 \longrightarrow N^{*}_{\varphi (x)}X_1, \end{aligned}$$

and, moreover, *F* satisfies $F^*(\omega _{\mathcal {V}_1})=\omega _{\mathcal {V}_0}$.

Let us remark that there are relatively few normal form results for Poisson maps in the literature (mostly for moment maps on symplectic manifolds [11, 15] and, in particular, for integrable systems [7, 8, 18]), and that our result is of a very different nature: it holds without any “compactness/properness assumptions”, which is just further evidence of the central role played by Poisson transversals in Poisson geometry.^1^

Next, we move to symplectic groupoids. As a general principle, which follows from the normal form theorem, Poisson transversals encode all the geometry of a neighborhood in the ambient manifold, and ’transverse properties’ should hold for the transversal if and only if they hold true around it. We show that integrability by a symplectic groupoid is one such transverse property:

### Theorem 2

(Integrability as a transverse property) A Poisson transversal is integrable if and only if it has an integrable open neighborhood.

In fact, we show much more:

### Theorem 3

(Normal form for symplectic groupoids) Let $(X,\pi _X)$ be a Poisson transversal in $(M,\pi )$, and consider a tubular neighborhood $M \supset E {\mathop {\rightarrow }\limits ^{p}} X$ in which the Poisson structure is in normal form, i.e., $\pi |_E=\pi _X^{\sigma }$. If $(X,\pi _X)$ is integrable by a symplectic groupoid $(\mathcal {G}_X,\omega _X) \rightrightarrows (X,\pi _X)$, then:

- A symplectic groupoid integrating $\pi _X^{\sigma }$ is $(\mathcal {G}_X^E,\omega _E) \rightrightarrows (E,\pi _X^{\sigma })$, where:
  $$\begin{aligned} \mathcal {G}_X^E:=\mathcal {G}_X \times _{\mathcal {P}(X)}\mathcal {P}(E), \ \ \ \ \omega _E:=\mathbf {p}^{*}(\omega _{X})+\mathbf {s}^{*}(\sigma )-\mathbf {t}^{*}(\sigma ). \end{aligned}$$
  Here $\mathcal {P}(\mathcal {M}) \rightrightarrows \mathcal {M}$ stands for the pair groupoid of a manifold $\mathcal {M}$, and $\mathbf {p}:\mathcal {G}_X^E \rightarrow \mathcal {G}_X$ stands for the canonical groupoid map.
- The restriction to *E* of any symplectic groupoid $(\mathcal {G},\omega _{\mathcal {G}}) \rightrightarrows (M,\pi )$ integrating $\pi$ is isomorphic to the model $(\mathcal {G}_X^E,\omega _E)$ corresponding to $\mathcal {G}_X:=\mathcal {G}|_X$, $\omega _X:=\omega _{\mathcal {G}}|_{\mathcal {G}_X}$.

We conclude the paper by illustrating our results in the setting of linear Poisson structures, i.e., Lie algebras. Although in this linear setting the conclusions of our results are well-known, the usage of Poisson transversals gives a new perspective on some classical results.

## Preliminaries on Poisson transversals

Recall from [10] that an embedded submanifold $X \subset M$ in a Poisson manifold $(M,\pi )$ is said to be a **Poisson transversal** if it induces a splitting:

2 $$\begin{aligned} TX \oplus NX=TM|_X, \end{aligned}$$

where $NX:=\pi ^{\sharp }(N^{*} X)\subset TM|_{X}$ will be called the **embedded normal bundle**. As explained in [10], the restriction $\pi |_{X}$ of $\pi$ to $T^{*}M|_X$ decomposes as:

$$\begin{aligned} \pi |_{X}=\pi _{X}+w_{X}, \end{aligned}$$

where $\pi _{X}\in {\varGamma }(\bigwedge ^2TX)$ is a Poisson structure and $w_X \in {\varGamma }(\bigwedge ^2N X)$ is a nondegenerate bivector. The main result of [10] is that pair $(\pi _X,w_X)$ encodes the structure of $\pi$ around *X*. To explain this, recall:

### Definition 1

Let $(M,\pi )$ be a Poisson manifold. A vector field $\mathcal {V}\in \mathfrak {X}(T^{*}M)$ is a **spray** for $\pi$ if:

1. $$m_t^{*}(\mathcal {V})=t\mathcal {V}$$, for all $$t>0$$;
2. $${\text {pr}}_{*}\mathcal {V}(\xi )=\pi ^{\sharp }(\xi )$$, for all $$\xi \in T^{*}M$$,

where $m_t:T^{*}M \rightarrow T^{*}M$ denotes the map of scalar multiplication by *t*.

### Remark 1

A spray on a Poisson manifold $(M,\pi )$ can be easily constructed: e.g., the horizontal lift $\mathcal {V}(\xi )$ of $\pi ^{\sharp }(\xi )$ with respect to a fixed linear connection is a Poisson spray. More generally, a $T^*M$ Lie algebroid connection $\nabla$ on $T^*M$ [9] induces a $T^*M$-geodesic flow which comes from a spray $\mathcal {V}_{\nabla }$. In fact, it is easily seen that any spray comes from a $T^*M$-connection, and moreover, there is a unique such connection which is torsion-free. Thus, all our constructions can be done in terms of connection; however, we prefer the spray terminology.

The following result played a crucial role in the proof of the normal form theorem in [10]:

### Theorem A

[5] Let $\pi$ be Poisson and denote by $\phi _t$ the time-*t* (local) flow of a spray $\mathcal {V}$ for $\pi$. Then there is an open ${\varSigma }_{\mathcal {V}} \subset T^{*}M$ around *M* with the property that:

1. $\phi$ is defined on ${\varSigma }_{\mathcal {V}} \times [0,1]$;
2. The closed two-form ${\varOmega }_{\mathcal {V}}:=\int _0^1\phi _t^{*}\omega _{\mathrm {can}}dt$ is symplectic on ${\varSigma }_{\mathcal {V}}$;
3. The submersions
  $$\begin{aligned} (M,\pi ){\mathop {\longleftarrow }\limits ^{{\text {pr}}}}({\varSigma }_{\mathcal {V}},{\varOmega }_{\mathcal {V}}){\mathop {\longrightarrow }\limits ^{\exp _{\mathcal {V}}}}(M,-\pi ) \end{aligned}$$
  give a full dual pair, where $\exp _{\mathcal {V}}:={\text {pr}}\circ \phi _1$.

Let $X\subset (M,\pi )$ be a Poisson transversal with associated pair $(\pi _X,w_X)$. We denote by ${\varUpsilon }(w_X)$ the space of all closed two-forms $\sigma \in {\varOmega }^2(N^{*}X)$ which along *X* satisfy $\sigma |_X=-w_X\in {\varGamma }(\bigwedge ^2 NX)$, where we identify $\bigwedge ^2 NX$ with the space of vertical two-forms in $\bigwedge ^2 T^*(N^*X)|_X$. To each $\sigma \in {\varUpsilon }(w_X)$, there corresponds a local model of $\pi$ around *X*, which, in Dirac-geometric terms, is described as the Poisson structure $\pi _X^{\sigma }$ corresponding to the Dirac structure ${\text {pr}}^*(L_{\pi _X})^{\sigma }$. As shown in [10], $\pi _X^{\sigma }$ is defined in a neighborhood of *X* in $N^*X$, and for any other $\sigma ' \in {\varUpsilon }(w_X)$, $\pi _X^{\sigma }$ and $\pi _X^{\sigma '}$ are Poisson diffeomorphic around *X*, by a diffeomorphism that fixes *X* to first order.

### Theorem B

[10] In the notation of Theorem A, the two-form $\omega _{\mathcal {V}}:=-{\varOmega }_{\mathcal {V}}|_{N^{*}X}$ belongs to ${\varUpsilon }(w_X)$, and the exponential map yields a Poisson embedding around *X*,

$$\begin{aligned} \exp _{\mathcal {V}}:(N^{*}X,\pi _X^{\omega _{\mathcal {V}}}) \hookrightarrow (M,\pi ). \end{aligned}$$

### Remark 2

In Theorem B, $\exp _{\mathcal {V}}$, and $\omega _{\mathcal {V}}$ are defined only on small enough neighborhoods of *X* in $N^*X$, but we still write $\exp _{\mathcal {V}}:N^{*}X\rightarrow M$, and $\omega _{\mathcal {V}}\in {\varUpsilon }(w_X)$. This convention will be used throughout Sect. 3, also for other maps and tensors, as it simplifies notation considerably.

## Normal form for Poisson maps

The result below is a the first indication for a normal form theorem for Poisson maps should hold around Poisson transversals; we refer the reader to [10] for a proof:

### Lemma 1

Let $\varphi :(M_0,\pi _0)\rightarrow (M_1,\pi _1)$ be a Poisson map and $X_1\subset M_1$ be a Poisson transversal. Then:

1. $\varphi$ is transverse to $X_1$;
2. $X_0:=\varphi ^{-1}(X_1)$ is also a Poisson transversal;
3. $\varphi$ restricts to a Poisson map $\varphi |_{X_0}:(X_0,\pi _{X_0}) \rightarrow (X_1,\pi _{X_1})$;
4. The differential of $\varphi$ along $X_0$ restricts to a fiberwise linear isomorphism between embedded normal bundles $\varphi _*|_{NX_0}:NX_0\rightarrow NX_1$;
5. The map $F:N^*X_0\rightarrow N^*X_1$, $F(\xi )=(\varphi ^*)^{-1}(\xi )$, $\xi \in N^*X_0$ is a fiberwise linear symplectomorphism between the symplectic vector bundles
  $$\begin{aligned}F:(N^*X_0,w_{X_0})\rightarrow (N^*X_1,w_{X_1}).\end{aligned}$$

We are ready to state the main result of this section. Consider the same setting as in Lemma 1.

### Theorem 4

(Normal form for Poisson maps) There are sprays $\mathcal {V}_0$ for $\pi _0$, and $\mathcal {V}_1$ for $\pi _1$, such that under the induced exponentials $\exp _{\mathcal {V}_i}:(N^{*}X_i,\pi _{X_i}^{\omega _{\mathcal {V}_i}}) \hookrightarrow (M_i,\pi _i)$, the map $\varphi$ corresponds to the bundle map *F*, and $F^*(\omega _{\mathcal {V}_1})=\omega _{\mathcal {V}_0}$. In particular, we have a commutative diagram of Poisson maps:


In other words, the theorem allows us to bring simultaneously both Poisson structures in normal form and the Poisson map becomes linear in the normal directions. This specializes to the normal form theorem of [10] by taking $M_0=M_1$, $X_0=X_1$ and $\varphi ={\text {id}}$. Remark 2 applies also here: the result is only local around $X_0$ and $X_1$, as the exponential maps $\exp _{\mathcal {V}_i}$ are defined only around $X_i$. Moreover, if $X_i$ are not closed submanifolds, then we can only guarantee that the sprays $\mathcal {V}_i$ are defined around $X_i$.

### Proof (of Theorem 4)

We split the proof into five steps:

*Step 1: Extending the map* *F* *around* $X_0$ *and* $X_1$. Let $U_1\subset M_1$ be an open neighborhood of $X_1$, so small that there exists a subbundle $H_1\subset TU_1$ to which $\varphi$ is transverse, and which satisfies $H_1|_{X_1}=TX_1$. Define

$$\begin{aligned} U_0:=\varphi ^{-1}(U_1)\subset M_0, \ \ H_0:=(\varphi _*)^{-1}(H_1)\subset TM_0. \end{aligned}$$

The fact that $\varphi$ is transverse to $H_1$ implies that $H_0$ is a smooth subbundle. Consider the annihilators of the distributions $H_0$ and $H_1$:

$$\begin{aligned} C^*_0:=(H_0)^{\circ }\subset T^*U_0, \ \ C^*_1:=(H_1)^{\circ }\subset T^*U_1. \end{aligned}$$

Transversality also implies that, for every $x\in U_0$, the transposed of the differential restricts to a linear isomorphism between the annihilators:The fiberwise inverse of this map gives the vector bundle map, denoted3which extends the map *F*.

*Step 2: Constructing* $\widetilde{F}$ *-related sprays on* $C^*_0$ *and* $C^*_1$. A vector bundle $E\rightarrow M$ endowed with a linear map $E\rightarrow TM$ will be called an anchored vector bundle. Note that $\pi _i^{\sharp }:C^*_i\rightarrow TU_i$ are anchored vector bundles, for $i=0,1$. Moreover, that $\varphi$ is a Poisson map implies that $\widetilde{F}$ is a map of anchored vector bundles:

4 $$\begin{aligned} \varphi _*\pi _0^{\sharp }(\xi )=\varphi _*\pi _0^{\sharp }\varphi ^*(\widetilde{F}\xi )=\pi _1^{\sharp }(\widetilde{F}\xi ). \end{aligned}$$

Next, the notion of spray has an obvious generalization to any anchored vector bundle. Let $\mathcal {V}_1\in \mathfrak {X}(C^*_1)$ be a spray on $C^*_{1}$. Since (3) forms a pullback diagram, we can pullback $\mathcal {V}_1$ to a spray $\mathcal {V}_0$ on the anchored vector bundle $C^*_0$. Indeed, for all $x\in U_0$ and $\xi \in C_{0,x}^*$, the differentials form a pullback diagram:and therefore, there is a unique vector $\mathcal {V}_0(\xi )\in T_{\xi }C_0^*$ satisfying:

$$\begin{aligned} {\text {pr}}_*(\mathcal {V}_0(\xi ))=\pi _0^\sharp (\xi ), \ \ \ \widetilde{F}_*(\mathcal {V}_0(\xi ))=\mathcal {V}_1(\xi ); \end{aligned}$$

existence follows from (4). Clearly, $\mathcal {V}_0$ and $\mathcal {V}_1$ are $\widetilde{F}$-related and $\mathcal {V}_0$ satisfies the second spray condition. The first spray condition follows by noting that also $\frac{1}{t}(m_{t,*})^{-1}(\mathcal {V}_0(t\xi ))$ satisfies the same equations as $\mathcal {V}_0(\xi )$.

*Step 3: Extending the sprays.* Let $\rho :E\rightarrow TN$ be an anchored vector bundle, let $F\subset E$ be a vector subbundle, and let $\mathcal {V}$ be a spray on *F*. Consider a complement $E=F\oplus G$, and linear connections on *F* and *G* with horizontal lifts denoted $h^F$ and $h^G$, respectively. Using the canonical isomorphism $TE\simeq TF\times _{TN}TG$, we construct the following spray $\widetilde{\mathcal {V}}$ on *E*:

$$\begin{aligned} \widetilde{\mathcal {V}}(f,g)=(\mathcal {V}(\rho (f))+h^F(\rho (g)),h^G(\rho (f)+\rho (g)))\in T_fF\times T_gG. \end{aligned}$$

Moreover, note that $\widetilde{\mathcal {V}}$ is tangent to *F* and extends $\mathcal {V}$.

Applying this construction, find sprays $\widetilde{\mathcal {V}}_i$ on $T^*U_i$, for $i=0,1$, which are tangent to $C^*_i$, and extend $\mathcal {V}_i$. Note that, if $X_i$ is a closed submanifold of $M_i$, then $\widetilde{\mathcal {V}}_i$ can be extended to the entire $T^*M_i$.

To simplify notation, we will denote $\widetilde{\mathcal {V}}_i$ also by $\mathcal {V}_i$.

*Step 4: Commutativity of the diagrams.* Let ${\varPhi }_{\mathcal {V}_i}^t$ denote the time-*t* local flow of $\mathcal {V}_i$. Since $\mathcal {V}_i$ is tangent to $C^*_i$, and $\widetilde{F}_{*}\mathcal {V}_0=\mathcal {V}_1$, on $C^*_0$ we have that $\widetilde{F}\circ {\varPhi }_{\mathcal {V}_0}^t={\varPhi }_{\mathcal {V}_1}^t\circ \widetilde{F}$. Since $\widetilde{F}$ extends *F*, we obtain the commutative diagram:which implies the equality $\varphi \circ \exp _{\mathcal {V}_0}=\exp _{\mathcal {V}_1}\circ F$ from the statement.

*Step 5: Compatibility of the two-forms.* As in Theorems A and B, we denote by ${\varOmega }_{\mathcal {V}_i}:=\int _0^1({\varPhi }^t_{\mathcal {V}_i})^{*}\omega _{\mathrm {can}}dt$ and $\omega _{\mathcal {V}_i}:=-{\varOmega }_{\mathcal {V}_i}|_{N^*X_i}$. By Theorem B, the exponentials $\exp _{\mathcal {V}_i}:(N^*X_i,\pi _{X_i}^{\omega _{\mathcal {V}_i}})\hookrightarrow (M_i,\pi _i)$ are Poisson diffeomorphisms around $X_i$. Hence, also $F:(N^*X_0,\pi _{X_0}^{\omega _{\mathcal {V}_0}})\rightarrow (N^*X_1,\pi _{X_1}^{\omega _{\mathcal {V}_1}})$ is a Poisson map in a neighborhood of $X_0$. This does not directly imply that $F^*(\omega _{\mathcal {V}_1})=\omega _{\mathcal {V}_0}$, and this is what we prove next.

Recall that the tautological one-form $\lambda _{\mathrm {can}}\in {\varOmega }^1(T^*M_i)$ is defined by $\lambda _{\mathrm {can},\xi }(v):=\langle \xi ,{\text {pr}}_*(v)\rangle$, for $\xi \in T^*M_i$ and $v\in T_{\xi }(T^*M_i)$. We show now that $\widetilde{F}$ satisfies: $\widetilde{F}^*(\lambda _{\mathrm {can}}|_{C^*_1})=\lambda _{\mathrm {can}}|_{C^*_0}$. For $\xi \in C^*_0$ and $v\in T_{\xi }C^*_0$, we have:

$$\begin{aligned} (\widetilde{F}^*\lambda _{\mathrm {can}})_{\xi }(v)&= \langle \widetilde{F}(\xi ),{\text {pr}}_*(\widetilde{F}_*(v))\rangle =\langle (\varphi ^*)^{-1}(\xi ),({\text {pr}}\circ \widetilde{F})_*(v)\rangle \\&=\langle (\varphi ^*)^{-1}(\xi ),(\varphi \circ {\text {pr}})_*(v)\rangle =\langle (\varphi ^*)^{-1}(\xi ),\varphi _*({\text {pr}}_*(v))\rangle \\&=\langle \xi ,{\text {pr}}_*(v)\rangle =\lambda _{\mathrm {can},\xi }(v). \end{aligned}$$

This implies that $\widetilde{F}^*(\omega _{\mathrm {can}}|_{C^*_1})=\omega _{\mathrm {can}}|_{C^*_0}$. Using that $\widetilde{F}$ intertwines the flows of the sprays, and that these flows preserve the submanifolds $C^*_0$, $C^*_1$, we obtain:

$$\begin{aligned} ({\varPhi }_{\mathcal {V}_0}^{t*}\omega _{\mathrm {can}})|_{C^*_0}&={\varPhi }_{\mathcal {V}_0}^{t*}(\omega _{\mathrm {can}}|_{C^*_0})={\varPhi }_{\mathcal {V}_0}^{t*}\circ \widetilde{F}^*(\omega _{\mathrm {can}}|_{C^*_1})\\&=\widetilde{F}^*\circ {\varPhi }_{\mathcal {V}_1}^{t*}(\omega _{\mathrm {can}}|_{C^*_1})=\widetilde{F}^*({\varPhi }_{\mathcal {V}_1}^{t*}(\omega _{\mathrm {can}})|_{C^*_1}). \end{aligned}$$

Integrating for $t\in [0,1]$, this yields that ${\widetilde{F}^*({\varOmega }_{\mathcal {V}_1}|_{C^*_1})={\varOmega }_{\mathcal {V}_0}|_{C^*_0}}$ holds around $X_0$. Restricting to $N^*X_0$, we obtain the conclusion $F^*(\omega _{\mathcal {V}_1})=\omega _{\mathcal {V}_0}$. $\square$

## Integrability

Symplectic groupoids are the natural objects integrating Poisson manifolds. In this section, we discuss the relation between integrability of a Poisson manifold and integrability of one of its transversals. For integrable Poisson manifolds, we give a normal form theorem for the symplectic groupoid around its restriction to a Poisson transversal.

### Symplectic groupoids

We recollect here a few facts about symplectic groupoids and integrability of Poisson manifolds. For references, see [3, 4].

We denote the source/target maps of a Lie groupoid $\mathcal {G}\rightrightarrows M$ by $\mathbf {s},\mathbf {t}:\mathcal {G}\rightarrow M$, and the multiplication by $\mathbf {m}:\mathcal {G}\times _{\mathbf {s},\mathbf {t}}\mathcal {G}\rightarrow \mathcal {G}$.

A differential form $\eta \in {\varOmega }^q(\mathcal {G})$ is called **multiplicative** if

$$\begin{aligned} \mathbf {m}^{*}\eta ={\text {pr}}_1^{*}\eta +{\text {pr}}_2^{*}\eta \in {\varOmega }^q(\mathcal {G}\times _{\mathbf {s},\mathbf {t}}\mathcal {G}), \end{aligned}$$

where ${\text {pr}}_1, {\text {pr}}_2:\mathcal {G}\times _{\mathbf {s},\mathbf {t}}\mathcal {G}\rightarrow \mathcal {G}$ are the projections.

A **symplectic groupoid** is a Lie groupoid $\mathcal {G}\rightrightarrows M$ endowed with a multiplicative symplectic structure $\omega \in {\varOmega }^2(\mathcal {G})$. The base *M* of a symplectic groupoid $(\mathcal {G},\omega )$ carries a Poisson structure $\pi$ such that:

$$\begin{aligned} (M,\pi ){\mathop {\longleftarrow }\limits ^{\mathbf {s}}}(\mathcal {G},\omega ){\mathop {\longrightarrow }\limits ^{\mathbf {t}}}(M,-\pi ) \end{aligned}$$

is a full dual pair.

A Poisson manifold $(M,\pi )$ is called **integrable** if such a symplectic groupoid $(\mathcal {G},\omega )$ exists giving rise to $\pi$, in which case the groupoid is said to **integrate** $(M,\pi )$.

#### Theorem 5

A Poisson transversal $(X,\pi _X)$ of a Poisson manifold $(M,\pi )$ is integrable if and only if the restriction $(U,\pi |_U)$ of $\pi$ to an open neighborhood *U* of *X* is an integrable Poisson manifold.

#### Proof

Step 1: If. Let $({\varSigma },{\varOmega })\rightrightarrows (U,\pi )$ be a symplectic groupoid, and $p:U \supset E\rightarrow X$ be a tubular neighborhood on which the normal form holds: $\pi |_E=\pi _X^{\sigma }$, for some closed two-form $\sigma$ on *E*, satisfying $\sigma (v)=0$ for all $v\in TX$. Denote $\mathcal {G}_X:={\varSigma }|_X$, $\omega _X:={\varOmega }|_{\mathcal {G}_X}$. Then $\pi _X$ is integrable by the symplectic groupoid $(\mathcal {G}_X,\omega _X)\rightrightarrows (X,\pi _X)$. This is proved in [4], in the more general setting of “Lie-Dirac submanifolds” (Theorem 9); for completeness, we include a simple proof:

Applying Lemma 1 to the Poisson map

$$\begin{aligned} (\mathbf {t},\mathbf {s}): ({\varSigma },{\varOmega }) \rightarrow (U,-\pi )\times (U,\pi ), \end{aligned}$$

and the Poisson transversal $X \times X \subset U \times U$, we deduce that $(\mathbf {t},\mathbf {s})$ is transverse to $X \times X$, that $(\mathbf {t},\mathbf {s})^{-1}(X \times X)=:\mathcal {G}_X \subset {\varSigma }$ is a Poisson transversal in ${\varSigma }$ (thus $\omega _X$ is symplectic), and that the induced map

$$\begin{aligned} (\mathbf {t},\mathbf {s}): (\mathcal {G}_X,\omega _X) \rightarrow (X,-\pi _X)\times (X,\pi _X) \end{aligned}$$

is again Poisson. Hence, $(\mathcal {G}_X,\omega _X)$ is a symplectic groupoid integrating $(X,\pi _X)$.

Step 2 : Only if. Recall [14] that integrability of a Poisson manifold by a symplectic groupoid is equivalent to integrability of its cotangent Lie algebroid. In particular, $\mathcal {G}_X$ integrates $T^*X$. By Theorem B and Lemma 2 below, in a tubular neighborhood $p:E\rightarrow X$ of the Poisson transversal $(X,\pi _X)\subset (M,\pi )$, the cotangent Lie algebroid $T^*E$ of $\pi |_E$ is isomorphic to the pullback Lie algebroid $TE\times _{TX}T^*X$ of the cotangent Lie algebroid $T^*X$ of $\pi _X$ by *p*. By Proposition 1.3 [13], the pullback Lie algebroid $TE\times _{TX}T^*X$ is integrable by the pullback groupoid (see below), and so $(E,\pi _X^{\sigma })$ is integrable. $\square$

An inconvenient feature of both Theorem 5 and its proof is that we are left with a poor understanding of how the symplectic groupoids integrating $(X,\pi _X)$ and a neighborhood of it are related. This is the issue we address in the next section.

## Normal form for symplectic groupoids

Our next goal is to state and prove Theorem 6 below, which refines Theorem 5 in that it gives a precise description of the symplectic groupoid integrating a neighborhood of a Poisson transversal in terms of the symplectic groupoid integrating the Poisson transversal itself.

We begin with a description of the Lie algebroid structure corresponding to Poisson structures constructed using the ’Poisson transversal recipe’. Concretely, consider the following set-up, which appears around Poisson transversals:

- $(X,\pi _X)$ is a Poisson manifold;
- $p:E\rightarrow X$ is a surjective submersion;
- $\sigma$ is a closed two-form on *E* such that the Dirac structure $p^*(L_{\pi _X})^{\sigma }$ corresponds to a globally defined Poisson structure $\pi _X^{\sigma }$ on *E*.

Consider the pullback of the Lie algebroid $T^*X$ via the submersion $p:E\rightarrow X$ (see e.g., [12] for the general construction of Lie algebroid pullbacks)

$$\begin{aligned} TE\times _{TX}T^*X=\{(U,\eta )\in TE\times T^*X : p_*(U)=\pi _X^{\sharp }(\eta )\}. \end{aligned}$$

The Lie algebroid $TE\times _{TX}T^*X$ fits into a short exact sequence of Lie algebroids:

5 $$\begin{aligned} 0\longrightarrow \mathbb {V}\longrightarrow TE\times _{TX}T^*X\longrightarrow T^*X\longrightarrow 0, \end{aligned}$$

where $\mathbb {V}\subset TE$ denotes the Lie algebroid $\mathbb {V}=\ker (p_{*})$. We have:

### Lemma 2

The cotangent Lie algebroid $T^*E$ of $\pi _X^{\sigma }$ is isomorphic to the pullback Lie algebroid $TE\times _{TX}T^*X$ via the mapUnder this isomorphism, the short exact sequence (5) corresponds to

$$\begin{aligned} 0\longrightarrow \mathbb {V}{\mathop {\longrightarrow }\limits ^{\sigma ^{\sharp }}} T^*E \longrightarrow T^*X\longrightarrow 0, \end{aligned}$$

where the second map assigns to $\xi \in T^*E$ the unique $\eta \in T^*X$ for which $p^*(\eta )=\xi -\sigma ^{\sharp }((\pi _X^{\sigma })^{\sharp }(\xi ))$.

### Proof

We have a sequence of Lie algebroid isomorphisms: first, the cotangent Lie algebroid $T^*E$ of $\pi _X^{\sigma }$ is defined such that the mapbe a Lie algebroid isomorphism; next, the gauge transformation by $\sigma$ is also a Lie algebroid isomorphismand finally, the map , $U+\eta \mapsto U+p^*(\eta )$ is an isomorphism as well. The composition of these maps returns the morphisms from the statement. $\square$

We present next a general construction for symplectic groupoids, which provides the local model of a symplectic groupoid around its restriction to a Poisson transversal.

### A pullback construction for symplectic groupoids

The construction of the pullback groupoid is rather standard (according to [13], it dates back to Ehresmann). We reexamine the construction in the setting of symplectic groupoids, in order to obtain a more explicit proof of Theorem 5.

Let $\mathcal {P}(E):=E\times E\rightrightarrows E$ and $\mathcal {P}(X):=X\times X\rightrightarrows X$ stand, respectively, for the pair groupoids of *E* and *X*. Define the groupoid $\mathcal {G}_{X}^E\rightrightarrows E$ to be the pullback of the groupoid maps:6That is, $\mathcal {G}_X^E$ is the manifold

$$\begin{aligned} \mathcal {G}_{X}^E:=\left\{ (e',g,e): p(e')=\mathbf {t}(g), p(e)=\mathbf {s}(g) \right\} \subset E\times \mathcal {G}_X \times E, \end{aligned}$$

endowed with the structure maps

$$\begin{aligned}&\mathbf {s}(e',g,e)=e, \quad \mathbf {t}(e',g,e)=e', \quad (e'',h,e')(e',g,e)=(e'',hg,e)\\&\quad (e',g,e)^{-1}=(e,g^{-1},e'), \quad \mathbf {1}_e=(e,1_{p(e)},e); \end{aligned}$$

As pullbacks by groupoid maps of closed, multiplicative forms $\omega _X \in {\varOmega }^2(\mathcal {G}_X)$, $\sigma \in {\varOmega }^2(\mathcal {P}(E))$, both $\mathbf {p}^*(\omega _X)$ and $\mathbf {s}^*(\sigma )-\mathbf {t}^*(\sigma )$ are closed, multiplicative two-forms on $\mathcal {G}_X^E$, and hence so is their sum:

$$\begin{aligned} \omega _{E} \in {\varOmega }^2(\mathcal {G}_X^E), \qquad \omega _E:=\mathbf {p}^{*}(\omega _{X})+\mathbf {s}^{*}(\sigma )-\mathbf {t}^{*}(\sigma ). \end{aligned}$$

#### Proposition 1

$(\mathcal {G}_X^E,\omega _{E})\rightrightarrows (E,\pi _X^{\sigma })$ is a symplectic groupoid.

The proof of Proposition 1 uses some general remarks about Dirac structures and Dirac maps:

#### Lemma 3

Consider a commutative diagram of manifolds:where *A* and *TA* are identified with the set-theoretic pullbacks $A\cong B\times _DC$, and $TA\cong TB\times _{TD} TC$ (e.g., if $k:B\rightarrow D$ and $l:C\rightarrow D$ are transverse maps). Assume further that the manifolds above are endowed with Dirac structures: $L_A$ on *A*, $L_B$ on *B*, $L_C$ on *C*, and $L_D$ on *D*.

- If *k* and *i* are backward Dirac maps, and *l* is forward Dirac, then *j* is also forward Dirac.
- If $j:(A,L_A)\rightarrow (B,L_B)$ is forward Dirac, and $\omega$ is a closed two-form on *B*, then *j* is also a forward Dirac map between the gauge-transformed Dirac structures: $j:(A,L_A^{j^*(\omega )})\rightarrow (B,L_B^{\omega })$.
- If $L_A$ is the graph of a closed two-form $\omega$ on *A*, and $L_B$ is the graph of a Poisson structure $\pi$ on *B*, and $j:(A,L_{A})\rightarrow (B,L_{B})$ is forward Dirac, then $\ker (\omega )\subset \ker (j_{*})$.

#### Proof

(a) Observe that, counting dimensions, it suffices to show that $j_{*}L_{A} \subset L_B$. Fix then $a \in A$, and set $b:=j(a)$, $c:=i(a)$ and $d:=k(b)=l(c)$. To further simplify the notation, we also let $L_a:=L_{A,a}$, $L_b:=L_{B,b}$, $L_c:=L_{C,c}$, and $L_d:=L_{D,d}$.

Choose $X_B+\eta _B \in j_*(L_{a})$. This means that $X_B=j_*(X_A)$, for some vector $X_A$ with $X_A + j^*(\eta _B)\in L_{a}$. Since *i* is a backward Dirac map, there is a covector $\eta _C$ such that $j^*(\eta _B)=i^*(\eta _C)$ and $i_*(X_A)+\eta _C\in L_c$. Since $i^*(\eta _C)=j^*(\eta _B)$, the dual of the pullback property for *TA* implies that there is a covector $\eta _D\in T^*_dD$, with $\eta _C=l^*(\eta _D)$ and $\eta _B=k^*(\eta _D)$. Since *l* is a forward Dirac map, we have that $l_*(X_C)+\eta _D \in L_d$. Commutativity of the diagram implies that $l_*(X_C)=k_*(X_B)$. Thus $k_*(X_B)+\eta _D \in L_d$, and $k^*(\eta _D)=\eta _B$. Finally, since *k* is a backward Dirac map, $X_B + \eta _B \in L_b$. Hence $X_B+\eta _B \in L_b$, and the conclusion follows.

(b) Note that, again by dimensional reasons, we need only show that $L_b^{\omega }\subset j_*(L_a^{j^*(\omega )})$. Choose $a\in A$ and set $b:=j(a)$, $L_a:=L_{A,a}$ and $L_B:=L_{B,b}$. Consider $X_B + \eta _B \in L_b^{\omega }$. This means that $X_B + \eta _B-\iota _{X_B}\omega \in L_b$. Since *j* is a forward Dirac map, there is a vector $X_A$ with $X_B=j_*(X_A)$ and $X_A + j^*(\eta _B-\iota _{X_B}\omega )\in L_a$. Clearly, $j^*(\iota _{X_B}\omega )=j^*(\iota _{j_*(X_A)}\omega )=\iota _{X_A}j^*(\omega )$. Hence $X_A + j^*(\eta _B)-\iota _{X_A}j^*(\omega ) \in L_a$, and so $X_A+ j^*(\eta _B) \in L_a^{j^*(\omega )}$. This shows that $X_B + \eta _B \in j_*(L_a^{j^*(\omega )})$.

(c) If $V\in \ker (\omega )$, then $V \in L_{\omega }$. But *j* forward Dirac implies $j_*(V) \in L_{\pi }$, and therefore $j_*(V)=0$. $\square$

#### Proof (of Proposition 1)

We apply Lemma 3 (a) to the pullback diagram (6), where these manifolds have the following Dirac structures :

$$\begin{aligned}(X,-\pi _X)\times (X,\pi _X),\ (E, p^*(L_{-\pi _X}))\times (E, p^*(L_{\pi _X})),\ (\mathcal {G}_X,\omega _X),\ (\mathcal {G}_X^E, \mathbf {p}^*(\omega _X)).\end{aligned}$$

We deduce that the map

$$\begin{aligned} (\mathbf {t},\mathbf {s}):(\mathcal {G}_X^E,\mathbf {p}^*(\omega _X))\longrightarrow (E, p^*(L_{-\pi _X}))\times (E, p^*(L_{\pi _X})) \end{aligned}$$

is forward Dirac. By Lemma 3 (b), this map is forward Dirac also after gauge-transformations:

$$\begin{aligned} (\mathbf {t},\mathbf {s}):(\mathcal {G}_X^E, \omega _E)\longrightarrow (E,-\pi _X^{\sigma })\times (E,\pi _X^{\sigma }). \end{aligned}$$

It remains to show that $\omega _E$ is nondegenerate. As $\mathcal {G}_X^E=E\times _X\mathcal {G}_X\times _X E$, we obtain that its tangent bundle is the pullback $T\mathcal {G}_X^E=TE\times _{TX}T\mathcal {G}_X\times _{TX} TE$. Explicitly:

$$\begin{aligned} T\mathcal {G}_X^E=\{(A,B,C)\in TE\times T\mathcal {G}_X\times TE : p_{*}(A)=\mathbf {t}_{*}(B), \mathbf {s}_{*}(B)=p_{*}(C)\}. \end{aligned}$$

In this decomposition, we can write

7 $$\begin{aligned} \omega _E((A,B,C),(A',B',C'))=-\sigma (A,A')+\omega _X(B,B')+\sigma (C,C'). \end{aligned}$$

By Lemma 3 (c),

$$\begin{aligned} \ker (\omega _E)\subset \ker (\mathbf {s}_{*})\cap \ker (\mathbf {t}_{*})=\{(0,B,0) : \mathbf {s}_{*}(B)=0, \ \mathbf {t}_{*}(B)=0\} \end{aligned}$$

But, for $(0,B,0)\ne 0$ we have that $\iota _{(0,B,0)}\omega _E=\mathbf {p}^*(\iota _B\omega _X)\ne 0$, because $\omega _X$ is nondegenerate. Hence, $\omega _E$ is nondegenerate. Thus $(\mathcal {G}_X^E,\omega _E)$ is a symplectic groupoid integrating $(E,\pi _X^{\sigma })$. $\square$

### The normal form theorem

We are now ready to prove that the structure of a symplectic groupoid around a Poisson transversal is described by the pullback construction:

#### Theorem 6

(Normal form for symplectic groupoids) Let $({\varSigma },{\varOmega })\rightrightarrows (M,\pi )$ be a symplectic groupoid, and let $(X,\pi _X)$ be a Poisson transversal in *M*. Let $p:E\rightarrow X$ be a tubular neighborhood on which the normal form holds: $\pi |_E=\pi _X^{\sigma }$, for some closed two-form $\sigma$ on *E*, satisfying $\sigma ^{\sharp }(U)=0$ for all $U\in TX$. Denote

$$\begin{aligned} \mathcal {G}_X:={\varSigma }|_X,\ \ \omega _X:={\varOmega }|_{\mathcal {G}_X}, \ \ {\varSigma }_E:={\varSigma }|_E, \ \ {\varOmega }_E:={\varOmega }|_{{\varSigma }_E}. \end{aligned}$$

Then, the Lie algebroid isomorphism $TE\times _{TX}T^*X\cong T^*E$ of Lemma 2 integrates to an isomorphism of symplectic groupoids ${\varPsi }:(\mathcal {G}_X^E,\omega _E)\cong ({\varSigma }_E,{\varOmega }_E)$.

#### Proof

We split the proof into three steps: constructing ${\varPsi }$ as an isomorphism of Lie groupoids, showing that it is a symplectomorphism, and finally, that it integrates the isomorphism of Lie algebroids $TE\times _{TX}T^*X\cong T^*E$.

*Step 1: Construction of Lie groupoid isomorphism* ${\varPsi }$.

Let *A* denote the Lie algebroid of ${\varSigma }_E$, i.e.,

$$\begin{aligned} T{\varSigma }_E|_E=TE\oplus A, \ \ A=\ker (\mathbf {s}_{*}). \end{aligned}$$

The identification between the Lie algebroid *A* and the cotangent Lie algebroid $T^*E$ is obtained via the symplectic form:By Lemma 2, the map $\sigma ^{\sharp }:\mathbb {V}\rightarrow T^*E$ is an injective Lie algebroid morphism. Note that $\mathbb {V}$ is integrable by the submersion groupoid $E\times _XE\rightrightarrows E$ of $p:E\rightarrow X$. Since $E\times _XE$ has 1-connected $\mathbf {s}$-fibers, the Lie algebroid map

8 $$\begin{aligned} (-{\varOmega }_E^{\sharp })^{-1}\circ \sigma ^{\sharp }:\mathbb {V}\rightarrow A \end{aligned}$$

integrates to a Lie groupoid map

$$\begin{aligned} {\varPhi }:E\times _X E\longrightarrow {\varSigma }_E. \end{aligned}$$

For $e\in E$, denote by $\tau (e)\in E\times _X E$ the arrow that starts at $p(e)\in X\subset E$, and ends at *e*: $\tau (e):=(e,p(e))$, and define the map:

$$\begin{aligned} {\varPsi }: \mathcal {G}_X^E\longrightarrow {\varSigma }_E, \ \ {\varPsi }(e',g,e):={\varPhi }(\tau (e'))\cdot g\cdot {\varPhi }(\tau (e))^{-1}. \end{aligned}$$

It is straightforward to check that ${\varPsi }$ is an isomorphism of Lie groupoids, with inverse

$$\begin{aligned}&{\varTheta }: {\varSigma }_E\longrightarrow \mathcal {G}_X^E, \ \ {\varTheta }(\overline{g})=(e',{\varPhi }(\tau (e'))^{-1}\cdot \overline{g}\cdot {\varPhi }(\tau (e)),e),\\&\quad \text {where} \ \ \ e':=\mathbf {t}(\overline{g}), \ \ e:=\mathbf {s}(\overline{g}). \end{aligned}$$

*Step 2:* ${\varPsi }$ *is an isomorphism of symplectic groupoids.* We begin with the observation that the identification $TE\times _{TX} T\mathcal {G}_X\times _{TX}TE= T\mathcal {G}_X^E$ can be realized using the multiplication map:

$$\begin{aligned} TE\times _{TX} T\mathcal {G}_X\times _{TX}TE \ni (U,V,W)\mapsto \mathbf m _{*}\left( \mathbf m _{*}(\tau _{*}(U),V),\tau ^{-1}_{*}(W)\right) \in T\mathcal {G}_X^E. \end{aligned}$$

Therefore, for any multiplicative two-form $\eta$, we have that:

$$\begin{aligned}&\eta ((U,V,W),(U',V',W'))\\&\quad =\eta |_{E\times _XE}(\tau _{*}(U),\tau _{*}(U'))+\eta |_{\mathcal {G}_X}(V,V')+\eta |_{E\times _XE}(\tau ^{-1}_{*}(W),\tau ^{-1}_{*}(W')). \end{aligned}$$

Therefore, in order to prove that $\omega _E$ and $\widetilde{\omega }_E:={\varPsi }^*({\varOmega }_E)$ coincide, it suffices to show that they have the same restriction to the subgroupoids $\mathcal {G}_X,E \times _X E$, which is what we turn to next.

That $\omega _E|_{\mathcal {G}_X}=\widetilde{\omega }_E|_{\mathcal {G}_X}$ follows by our construction: indeed, we have ${\varPsi }|_{\mathcal {G}_X}=\text {id}$ and $\omega _X={\varOmega }_E|_{\mathcal {G}_X}$; since $\sigma |_X=0$, also $\omega _X=\omega _E|_{\mathcal {G}_X}$, and hence our conclusion.

We next show that $\omega _E|_{E\times _XE}=\widetilde{\omega }_E|_{E\times _XE}$. Regarding $E\times _XE$ as the subgroupoid of $\mathcal {G}_X^E$ consisting of elements $(e', 1_x, e)$, for $p(e')=x=p(e)$, we clearly have ${\varPsi }|_{E\times _XE}={\varPhi }$, and

$$\begin{aligned} \omega _E|_{E\times _XE}=\mathbf {s}^*(\sigma )-\mathbf {t}^*(\sigma ). \end{aligned}$$

Now, ${\varPhi }^*({\varOmega }_E)$ is a multiplicative two-form on the source-simply connected groupoid $E\times _XE$, and is thus determined by its IM-form [1]. The IM-form^2^ corresponding to ${\varOmega }_E$ is simply $-{\varOmega }_E^{\sharp }:A\rightarrow T^*E$. Pulling it back via the Lie algebroid map (8) to $\mathbb {V}$, we deduce that the IM-form corresponding to ${\varPhi }^*({\varOmega }_E)$ is $\sigma ^{\sharp }:\mathbb {V}\rightarrow T^*E$, which is also the IM-form of the multiplicative two-form $\mathbf s ^*(\sigma )-\mathbf t ^*(\sigma )$. We thus conclude that $\mathbf s ^*(\sigma )-\mathbf t ^*(\sigma )={\varPhi }^*({\varOmega }_E)$, that

$$\begin{aligned} \omega _E|_{E\times _XE}=\widetilde{\omega }_E|_{E\times _XE}, \end{aligned}$$

and that ${\varPsi }$ is an isomorphism of symplectic groupoids.

*Step 3:* ${\varPsi }$ *integrates the Lie algebroid isomorphism of Lemma* 2. Note that the algebroid of $\mathcal {G}_X$ is given by

$$\begin{aligned} T\mathcal {G}_X|_X=TX\oplus A_X, \ \ A_X:=\{v\in A: \mathbf t _{*}(v)\in TX\}\subset A|_X; \ \ \end{aligned}$$

and the identification of $A_X$ with the cotangent Lie algebroid of $\pi _X$ is given by . Now, the Lie algebroid of $\mathcal {G}_X^E$ is the pullback Lie algebroid $TE\times _{TX}A_X$. Let $\psi$ denote the Lie algebroid map induced by ${\varPsi }$. Consider the commutative diagram of Lie algebroid isomorphisms:where the top-right triangle is commutative by the fact that ${\varPsi }^*({\varOmega }_E)=\omega _E$, and $\varphi$ is defined such that the entire diagram is commutative, i.e.,

$$\begin{aligned} \varphi :=(\text {id},(-\omega _X)^{\sharp })\circ (-\omega _E^{\sharp })^{-1}. \end{aligned}$$

We need to check that $\varphi$ is the isomorphism from Lemma 2, and for that we need to compute $(\omega _E)^{\sharp }$. At a unit $e\in E$, with $x=p(e)$, there are two decompositions of the tangent space to $\mathcal {G}_X^E$:

$$\begin{aligned} T_{e}\mathcal {G}_X^E\cong T_eE\oplus T_eE\times _{T_xX}A_{X,x}\cong T_eE\times _{T_xX}T_x\mathcal {G}_X\times _{T_xX} T_eE. \end{aligned}$$

In the first decomposition, the first factor is the tangent space to the units, and the second is the Lie algebroid (i.e., the tangent space to the source-fiber), whereas the second decomposition is based on the pullback construction of $\mathcal {G}_X^E=E\times _X\mathcal {G}_X\times _X E$. The identification between these decompositions is given by:

$$\begin{aligned} (U,V,W)\mapsto (U+V,p_*(U)+W,U). \end{aligned}$$

Using the expression (7) of $\omega _E$ with respect to the second decomposition, and the identification above, we compute $\omega _E$ with respect to the first decomposition:

$$\begin{aligned} \omega _E((0,V,W),(U,0,0))&=\left( s^*(\sigma )-t^*(\sigma )+\mathbf {p}^*(\omega _X)\right) (V,W,0)(U,p_*(U),U)\\&=-\sigma (V,U)+\omega _X(W,p_*(U)); \end{aligned}$$

therefore:

$$\begin{aligned} (-\omega _E)^{\sharp }(V,W)=\sigma (V)-p^*((\omega _X)^{\sharp }(W)). \end{aligned}$$

This shows that the diagram commutes for $\varphi (V,\eta )=\sigma ^{\sharp }(V)+p^*(\eta )$, which is the map from Lemma 2. This finishes the proof. $\square$

## Linear Poisson structures

In this section, we write our results explicitly for linear Poisson structures. Our goal is to illustrate Theorems A, B, 1 and 3 in this context, thus recasting and reproving some well-known results in what (we would argue) is their proper setting.

Let $(\mathfrak {g},[\cdot ,\cdot ])$ be a Lie algebra. The dual vector space $\mathfrak {g}^*$ carries a canonical Poisson structure $\pi _{\mathfrak {g}}$, called the linear Poisson structure. It is defined by

$$\begin{aligned} \pi _{\mathfrak {g},\xi }:=\xi \circ [\cdot ,\cdot ]\in \wedge ^2\mathfrak {g}^*=\wedge ^2T_{\xi }\mathfrak {g}^*. \end{aligned}$$

In fact, any Poisson structure on a vector space for which the linear functions form a Lie subalgebra is of this form.

Linear Poisson structures are always integrable. The following construction of a symplectic groupoid integrating $(\mathfrak {g}^*,\pi _{\mathfrak {g}})$ is standard and we recall it to establish the notation. Let *G* be a Lie group integrating $\mathfrak {g}$. Then a symplectic groupoid integrating $\pi _{\mathfrak {g}}$ is the action groupoid:

$$\begin{aligned} \left( G\ltimes \mathfrak {g}^*,{\varOmega }_{G}\right) \rightrightarrows (\mathfrak {g}^*,\pi _{\mathfrak {g}}) \end{aligned}$$

associated with the coadjoint action $(g,\xi )\mapsto \mathrm {Ad}_{g^{-1}}^*\xi$; it carries the symplectic structure: ${\varOmega }_G\in {\varOmega }^2(G\times \mathfrak {g}^*)$ given by:

9 $$\begin{aligned} {\varOmega }_G((x,\xi ),(y,\eta ))_{(g,\xi _0)}=\xi (g^{-1}y)-\eta (g^{-1}x)+\xi _0([g^{-1}x,g^{-1}y]), \end{aligned}$$

for $(x,\xi ),(y,\eta )\in T_{(g,\xi _0)}(G\times \mathfrak {g}^*)=T_gG\times \mathfrak {g}^*$, where $g^{-1}x$ and $g^{-1}y$ denotes the action of *G* on *TG*. For a detailed exposition (with similar notation) see, e.g., [16, Section 2.4.2].

### Illustration 1

- The Poisson manifold $(\mathfrak {g}^*,\pi _{\mathfrak {g}})$ carries a canonical, complete Poisson spray $\mathcal {V}_{\mathfrak {g}}$, whose flow (under the identification $T^*\mathfrak {g}^*=\mathfrak {g}\times \mathfrak {g}^*$) is given by:
  $$\begin{aligned} \phi _t:T^*\mathfrak {g}^*\longrightarrow T^*\mathfrak {g}^*,\ \ (x,\xi )\mapsto (x,e^{-t\mathrm {ad}_x^*}\xi ). \end{aligned}$$
- Let $\mathcal {O}(\mathfrak {g}) \subset \mathfrak {g}$ be the subspace where the Lie-theoretic exponential map $\exp :\mathfrak {g} \rightarrow G$ is a local diffeomorphism. Then the closed two-form:
  $$\begin{aligned} {\varOmega }_{\mathfrak {g}}:=\int _0^1\phi _t^*\omega _{\mathrm {can}}\mathrm {d}t \ \ \in \ \ {\varOmega }^2(T^{*}\mathfrak {g}^{*}) \end{aligned}$$
  is symplectic exactly on $\mathcal {O}(\mathfrak {g}) \times \mathfrak {g}^* \subset T^*\mathfrak {g}^*$, and gives rise to the full dual pair:  Explicitly:
  10 $$\begin{aligned} {\varOmega }_{\mathfrak {g}}((x,\xi ),(y,\eta ))_{(x_0,\xi _0)}=\xi ({\varXi }_{x_0}y)-\eta ({\varXi }_{x_0}x)+\xi _0\left( \left[ {\varXi }_{x_0}x,{\varXi }_{x_0}y\right] \right) , \end{aligned}$$
  where ${\varXi }_{x_0}$ is the linear endomorphism of $\mathfrak {g}$ given by:
  $$\begin{aligned} {\varXi }_{x_0}(x)=\int _{0}^1e^{-t\mathrm {ad}_{x_0}}(x)\mathrm {d}t=\frac{e^{-\mathrm {ad}_{x_0}}-\mathrm {Id}_{\mathfrak {g}}}{-\mathrm {ad}_{x_0}}(x). \end{aligned}$$
- $X\subset \mathfrak {g}^*$ is a Poisson transversal if and only if, for every $\lambda \in X$, the two-form $\lambda \circ [\cdot ,\cdot ]$ is nondegenerate on the annihilator of $T_{\lambda }X$. Moreover, under the identification $N^*X=\bigcup _{\lambda \in X} N_{\lambda }^{*}X\times \{\lambda \}\subset \mathfrak {g}\times X$, we have a Poisson diffeomorphism in a neighborhood of *X* given by:
  $$\begin{aligned} \exp _{\mathcal {V}_{\mathfrak {g}}}:(N^*X,\pi _X^{-{\varOmega }_{\mathfrak {g}}|_{N^*X}})\rightarrow (\mathfrak {g}^*,\pi _{\mathfrak {g}}) \ \ (x,\lambda )\mapsto e^{-\mathrm {ad}_x^*}\lambda ; \end{aligned}$$
- If $f:\mathfrak {g} \rightarrow \mathfrak {h}$ is a Lie algebra map, and $Y \subset \mathfrak {h}^*$ is a Poisson transversal, then $X:=(f^*)^{-1}Y \subset \mathfrak {g}^*$ is a Poisson transversal, and *f* induces a bundle map *F* fitting into the commutative diagram of Poisson maps:

### Proof (of Illustration 1)

- The flow $\phi _t$ in the statement has infinitesimal generator the vector field $\mathcal {V}_{\mathfrak {g}}\in \mathfrak {X}^1(\mathfrak {g}\times \mathfrak {g}^*)$ given by:
  $$\begin{aligned} \mathcal {V}_{\mathfrak {g},(x,\xi )}:=(0,-\mathrm {ad}_x^*\xi )=(0,\pi _{\mathfrak {g},\xi }^{\sharp }x)\in \mathfrak {g}\times \mathfrak {g}^*=T_{x}\mathfrak {g}\times T_{\xi }\mathfrak {g}^*, \end{aligned}$$
  where $\mathrm {ad}_xy=-[x,y]$ since we use right invariant vector fields to define the Lie bracket, and this is clearly a spray.
- Since trajectories $\phi _t(x,\xi )$ of $\mathcal {V}_{\mathfrak {g}}$ are cotangent paths, they can be integrated to elements in the Lie groupoid, yielding a *groupoid exponential map*:
  $$\begin{aligned} \mathrm {Exp}_{\mathcal {V}_{\mathfrak {g}}}:T^*\mathfrak {g}^*\longrightarrow G\ltimes \mathfrak {g}^*, \ \ (x,\xi )\mapsto (\exp (x),\xi ), \end{aligned}$$
  where $\exp :\mathfrak {g} \rightarrow G$ denotes the *Lie-theoretic exponential map*.
  On the other hand, the *spray exponential map* $\exp _{\mathcal {V}_{\mathfrak {g}}}$, i.e., the composition of $\phi _1$ with the bundle projection $T^*\mathfrak {g}^* \rightarrow \mathfrak {g}^*$, becomes $\mathrm {Exp}_{\mathcal {V}_{\mathfrak {g}}}$ composed with the target map:
  $$\begin{aligned} \exp _{\mathcal {V}_{\mathfrak {g}}}(x,\xi )=e^{-\mathrm {ad}_x^*}\xi . \end{aligned}$$
  Now, the pullback by $\mathrm {Exp}_{\mathcal {V}_{\mathfrak {g}}}$ of the symplectic structure ${\varOmega }_G$ of (9) is given by the formula in Theorem A (see [4] for details); hence, the general considerations above imply that the two-form ${\varOmega }_{\mathfrak {g}}$ is given by:
  11 $$\begin{aligned} {\varOmega }_{\mathfrak {g}}=(\mathrm {Exp}_{\mathcal {V}_{\mathfrak {g}}})^*{\varOmega }_{G}. \end{aligned}$$
  This implies that ${\varOmega }_{\mathfrak {g}}$ is nondegenerate exactly on $\mathcal {O}(\mathfrak {g})\times \mathfrak {g}^*$, and that the following is a commutative diagram of Poisson maps: 12 The explicit formula (10) for ${\varOmega }_{\mathfrak {g}}$ is obtained by pulling back ${\varOmega }_G$ from (9), and we conclude with the observation that the linear endomorphism ${\varXi }_{x_0}:\mathfrak {g} \rightarrow \mathfrak {g}$ is the left translation of the differential of $\exp :\mathfrak {g}\rightarrow G$ at $x_0$, and therefore it is invertible precisely on $\mathcal {O}(\mathfrak {g})$.
- Consider an affine subspace $\lambda +L$ passing through a point $\lambda \in \mathfrak {g}^*$ and with direction a linear subspace $L\subset \mathfrak {g}^*$. Now, $\lambda +L$ is a Poisson transversal in a neighborhood of $\lambda$ if and only if the following condition is satisfied:
  13 $$\begin{aligned} \mathfrak {g}^*=L\oplus L^{\circ }\cdot \lambda , \ \ L^{\circ }\cdot \lambda :=\{X\cdot \lambda : X\in L^{\circ }\}; \end{aligned}$$
  equivalently:
  14 $$\begin{aligned} \lambda \circ [\cdot ,\cdot ]|_{L^{\circ }\times L^{\circ }} \text { is a nondegenerate 2-form on }L^{\circ }. \end{aligned}$$
  The remaining claims are immediate.
- The dual map $f^*:(\mathfrak {h}^*,\pi _{\mathfrak {h}})\longrightarrow (\mathfrak {g}^*,\pi _{\mathfrak {g}})$ to a Lie algebra map *f* is a Poisson map, hence by Lemma 1, $f^*$ is transverse to *X*, $Y:=(f^*)^{-1}(X)$ is a Poisson transversal in $\mathfrak {h}^*$, and $f^*$ restricts to a Poisson map
  $$\begin{aligned} f^*|_{Y}:(Y,\pi _{Y})\longrightarrow (X,\pi _{X}). \end{aligned}$$
  Moreover, *f* restricts to a linear isomorphism between the conormal spaces , for all $\mu \in Y$. The inverses  can be put together in a vector bundle map  covering $f^*: Y \rightarrow X$, which is fiberwise a linear isomorphism.
  We conclude by showing that the diagram in the statement commutes. Let $(y,\xi )\in N^*Y$. Then $F(y,\xi )=(x,f^*(\xi ))\in N^*X$, where *x* satisfies $y=f(x)$. For any $z\in \mathfrak {g}$, we have:
  $$\begin{aligned}&\exp _{\mathcal {V}_{\mathfrak {g}}}\left( F(y,\xi )\right) (z)=\exp _{\mathcal {V}_{\mathfrak {g}}}((x,f^*\xi ))(z)=\left( e^{-\mathrm {ad}_x^*}f^*\xi \right) (z)\\&\quad =\xi (f(e^{-\mathrm {ad}_x}z))=\xi (e^{-\mathrm {ad}_{f(x)}}f(z))=\xi (e^{-\mathrm {ad}_{y}}f(z))\\&\quad =f^*(e^{-\mathrm {ad}_y^*}\xi )(z)=f^*(\exp _{\mathcal {V}_{\mathfrak {g}}}(y,\xi ))(z), \end{aligned}$$
  where we have used that *f* is a Lie algebra map. Since $f^*$ and the vertical maps are Poisson maps, it follows that also *F* is Poisson around *Y*.

$$\square $$

Our next illustration concerns the specialization of Theorem 6 for Poisson transversals complementary to coadjoint orbits, in the particularly convenient setting where the coadjoint action is *proper* at the orbit.

### Illustration 2

Let $\mathfrak {g}$ be a Lie algebra satisfying the following *splitting condition* at $\lambda \in \mathfrak {g}^*$: there is a decomposition

15 $$\begin{aligned} \mathfrak {g}=\mathfrak {g}_{\lambda }\oplus c, \end{aligned}$$

where $\mathfrak {g}_{\lambda }$ the isotropy Lie algebra at $\lambda$, satisfying $[\mathfrak {g}_{\lambda },c]\subset c$. Then:

- Along $\widetilde{X}:=\lambda +\mathfrak {g}_{\lambda }^*$, the Poisson tensor $\pi _{\mathfrak {g}}$ decomposes as:
  16 $$\begin{aligned} (\lambda +\xi )\circ \pi _{\mathfrak {g}}=\xi \circ \pi _{\mathfrak {g}_{\lambda }}+(\lambda +\xi )\circ \pi _c\in \wedge ^2\mathfrak {g}_{\lambda }^*\oplus \wedge ^2c^* \end{aligned}$$
  where we identify $\mathfrak {g}_{\lambda }^*=c^{\circ }$;
- $\widetilde{X}$ intersects all coadjoint orbits cleanly and symplectically, and hence inherits an induced Poisson structure $\pi _{\widetilde{X}}$;
- $\pi _{\widetilde{X}}$ is globally linearizable through the Poisson isomorphism:
- The subspace $X \subset \widetilde{X}$ where $\widetilde{X}$ is a Poisson transversal contains $\lambda$, and for a product neighborhood of the origin $V\times W\subset c\times \mathfrak {g}^*_{\lambda }$, the following map is an open Poisson embedding onto a neighborhood of $\lambda$:
  17 $$\begin{aligned} \left( V\times W, \pi _{\mathfrak {g}_{\lambda }}^{\sigma _{\lambda }}\right) \hookrightarrow \left( \mathfrak {g}^*,\pi _{\mathfrak {g}}\right) , \ \ (x,\xi )\mapsto e^{-\mathrm {ad}_x^*}(\lambda +\xi ), \end{aligned}$$
  where $\sigma _{\lambda }$ is the pullback of $-{\varOmega }_{\mathfrak {g}}$ via the map:
  $$\begin{aligned} c\times \mathfrak {g}^*_{\lambda }\rightarrow \mathfrak {g}\times \mathfrak {g}^*, \ \ (x,\xi )\mapsto (x,\lambda +\xi ); \end{aligned}$$
- If a Lie group *G* integrating $\mathfrak {g}$ acts properly at $\lambda$, and $G_{\lambda }$ denotes the isotropy group at $\lambda$, then, by shrinking $W\subset \mathfrak {g}_{\lambda }^*$ if need be, the restriction of the symplectic groupoid $G\ltimes \mathfrak {g}^*$ to the image of the map (17) is isomorphic to the product of the groupoid $G_{\lambda }\ltimes W\rightrightarrows W$ with the pair groupoid $V\times V\rightrightarrows V$, with symplectic structure:
  $$\begin{aligned}&\left( V\times \left( G_{\lambda }\ltimes W\right) \times V, \mathbf {s}^*(\sigma _{\lambda })+\mathbf {p}^*({\varOmega }_{G_{\lambda }})-\mathbf {t}^*(\sigma _{\lambda })\right) \rightrightarrows \left( V\times W,\pi _{\mathfrak {g}_{\lambda }}^{\sigma _{\lambda }}\right) ,\ \ \text {where}\\&\quad \mathbf {s}(y,(g,\xi ),x)=(x,\xi ), \ \ \mathbf {p}(y,(g,\xi ),x)=(g,\xi ), \ \ \mathbf {t}(y,(g,\xi ),x)=(y,\mathrm {Ad}_{g^{-1}}^*\xi ). \end{aligned}$$

### Remark 3

It was first proved in [17] that the splitting condition (15) implies that the transverse Poisson structure to the coadjoint orbit at $\lambda$ is linearizable, see also [23].

Submanifolds which intersect the symplectic leaves cleanly and symplectically, and for which the induced bivector is smooth, are called **Poisson-Dirac** [4]. In fact, the affine submanifold $\lambda +\mathfrak {g}_{\lambda }^*$ turns out to be a **Lie-Dirac** submanifold (also called “Dirac submanifold”), see [24, Example 2.18].

### Proof (of Illustration 2)

Since $[\mathfrak {g}_{\lambda },\mathfrak {g}_{\lambda }]\subset \mathfrak {g}_{\lambda }$ and $[\mathfrak {g}_{\lambda },c]\subset c$ we have that, in the decomposition:

$$\begin{aligned} \pi _{\mathfrak {g}}=\pi _{\mathfrak {g}_{\lambda }}+\pi _{m}+\pi _c, \end{aligned}$$

corresponding to (16), the components satisfy:

$$\begin{aligned} \pi _{\mathfrak {g}_{\lambda }}\in \mathfrak {g}_{\lambda }\otimes \wedge ^2 \mathfrak {g}_{\lambda }^*, \ \ \ \ \pi _{m}\in c\otimes (\mathfrak {g}_{\lambda }^*\otimes c^*), \ \ \ \ \pi _c\in \mathfrak {g}\otimes \wedge ^2 c^*. \end{aligned}$$

The fact that $\mathfrak {g}_{\lambda }$ is precisely the isotropy Lie algebra at $\lambda$ is equivalent to:

18 $$\begin{aligned} \lambda \circ \pi _{\mathfrak {g}_{\lambda }}=\lambda \circ \pi _m=0, \ \ \lambda \circ \pi _{c}\in \wedge ^2c^* \text { is nondegenerate}. \end{aligned}$$

Hence, on the affine space $\widetilde{X} = \lambda +\mathfrak {g}_{\lambda }^*$ the Poisson bivector takes the form (16). This proves (a), from which (b) and (c) follow.

The claim in (d) that $X \subset \widetilde{X}$ contains $\lambda$ is immediate. Write $X = \lambda +U$, where

$$\begin{aligned} U:=\left\{ \xi \in \mathfrak {g}_{\lambda }^* : (\lambda +\xi )\circ \pi _{c}\in \wedge ^2c^* \text { is nondegenerate}\right\} \subset \mathfrak {g}_{\lambda }^*. \end{aligned}$$

Observe that $N^{*}X=c\times X$ and, by part a), $NX=c^*\times X$. We thus recognize in (16) the decomposition (2) of $\pi _{\mathfrak {g}}$ along the Poisson transversal *X* into tangential and normal components. The remaining claim in d) is the conclusion of Theorem B around $\lambda$, for a product neighborhood $V\times W\subset c\times \mathfrak {g}^*_{\lambda }$ of the origin with $W\subset U$.

As for (e), note that the properness assumption implies that the group $G_{\lambda }$ is compact, that the coadjoint orbit through $\lambda$ is closed, and that the splitting (15) can be assumed to be $G_{\lambda }$-invariant. This assumption not only implies that the transverse Poisson structure is linearizable, but also that the Poisson manifold $(\mathfrak {g}^*,\pi _{\mathfrak {g}})$ is linearizable around the coadjoint orbit through $\lambda$ in the sense of [21], see [6, Example 2.7].

By the slice theorem for proper group actions, one can assume (by shrinking $W\subset \mathfrak {g}_{\lambda }^*$ if need be) that $\lambda +W$ is $G_{\lambda }$-invariant, and that its saturation is *G*-equivariantly diffeomorphic to $G\times _{G_{\lambda }}W$ via the map $[g,\xi ]\mapsto \mathrm {Ad}_{g^{-1}}^*(\lambda +\xi )$. In particular, this implies that the restriction of the action groupoid $G\ltimes \mathfrak {g}^*$ to $\lambda +W$ is isomorphic to the restriction of the action groupoid $G_{\lambda }\ltimes \mathfrak {g}_{\lambda }^*$ to *W*. This holds moreover at the level of symplectic groupoids, and the isomorphism is given by:The fact that the restriction of ${\varOmega }_{G}$ is ${\varOmega }_{G_{\lambda }}$ can be easily checked using (9) and (18).

A direct application of Theorem 6 now shows that the restriction of the symplectic groupoid $G\ltimes \mathfrak {g}^*$ to the image of the map (17) is isomorphic to the symplectic groupoid from e). $\square$

Recall that a Lie algebra $\mathfrak {h}$ is called a **Frobenius Lie algebra** if the coadjoint orbit through some $\lambda \in \mathfrak {h}^*$ is open.

### Illustration 3

Let $\mathfrak {h}$ be a Frobenius subalgebra of a Lie algebra $\mathfrak {g}$, and let $H \subset G$ be connected Lie groups integrating $\mathfrak {h} \subset \mathfrak {g}$. Denote by:

$$\begin{aligned} r:(\mathfrak {g}^*,\pi _{\mathfrak {g}})\longrightarrow (\mathfrak {h}^*,\pi _{\mathfrak {h}}) \end{aligned}$$

the Poisson submersion dual to the inclusion, and by $S \subset (\mathfrak {h}^*,\pi _{\mathfrak {h}})$ the (open) symplectic leaf through $\lambda \in \mathfrak {h}^*$.

- There is an open neighborhood $\mathcal {U}$ of 0 in $\mathfrak {h}$ such that the two-form on $\mathfrak {h}$
  $$\begin{aligned} \omega _{\lambda ,x_0}(x,y)=-\lambda \left( [{\varXi }_{x_0}x,{\varXi }_{x_0}y]\right) , \end{aligned}$$
  is nondegenerate on $\mathcal {U}$, and the map:
  19 $$\begin{aligned} (\mathcal {U},\omega _{\lambda }^{-1})\longrightarrow (\mathfrak {h}^*,\pi _{\mathfrak {h}}), \ \ x\mapsto e^{-\mathrm {ad}_x^*}\lambda \end{aligned}$$
  is a Poisson diffeomorphism onto a neighborhood of $\lambda$ in $\mathfrak {h}^*$;
- Around the Poisson transversal $X_{\lambda }:=r^{-1}(\lambda )$ there is a global Weinstein splitting of $\pi _{\mathfrak {g}}$ given by the commutative diagram of Poisson maps: 20
- Theorem 6 for $X_{\lambda }$ implies that the restriction of the symplectic groupoid $(G\ltimes \mathfrak {g}^*,{\varOmega }_G)$ to the image of (20) is isomorphic to the product of the symplectic groupoid $(G\ltimes \mathfrak {g}^*,{\varOmega }_G)|_{X_{\lambda }}$ and the symplectic pair groupoid $(\mathcal {U}\times \mathcal {U},\mathrm {pr}_1^*\omega _{\lambda }-\mathrm {pr}_2^*\omega _{\lambda })$;
- The Lie-theoretic exponential of *H*, $\exp :\mathfrak {h}\rightarrow H$, induces a factorization of diagram (20) through the commutative diagram of Poisson maps: 21 where $\widetilde{\lambda } \in {\varOmega }^1(H)$ is the left-invariant one-form extending $\lambda$, $H_{\lambda }$ is the stabilizer of $\lambda$, and the horizontal arrows are *H*-equivariant Poisson diffeomorphisms.

Part (d) gives a global description of the Poisson structure on the open $\mathfrak {g}^*|_S$, which implies the following:

### Corollary 1

The Poisson structure on $\mathfrak {g}^*|_S$ is horizontally nondegenerate for the submersion:

22 $$\begin{aligned} r:(\mathfrak {g}^*|_{S},\pi _{\mathfrak {g}})\longrightarrow (S,\pi _{\mathfrak {h}}). \end{aligned}$$

The corresponding Vorobjev triple (see, e.g., [21]) satisfies the following properties:

1. The horizontal distribution is involutive, and is given by the tangent bundle to the *H*-orbits;
2. The horizontal two-form is the pullback of the symplectic form on the leaf *S*;
3. In the decomposition $\pi _{\mathfrak {g}}|_{r^{-1}(S)}=\pi ^{\mathrm {v}}+\pi ^{\mathrm {h}}$ into vertical and horizontal components, we have that both bivectors are Poisson and commute.

### Remark 4

Note that, in general, the open set $\mathfrak {g}^*|_S$ is not saturated; for example, if $\mathfrak {h}$ is the diagonal subalgebra in $\mathfrak {g}=\mathfrak {aff}(1)\oplus \mathfrak {aff}(1)$.

We note also the following surprising property:

### Corollary 2

The induced Poisson structure $\pi _{X_{\mu }}$ on the Poisson transversal $X_{\mu }$ is at most quadratic for the canonical $\mathfrak {h}^{\circ }$-affine space structure on $X_{\mu }$.

### Remark 5

A special case of this corollary appeared in [19] when considering the transverse Poisson structure to the coadjoint orbit through an element $\xi \in \mathfrak {g}^*$ for which the isotropy Lie algebra $\mathfrak {g}_{\xi }$ has a complement $\mathfrak {h}$ which is also a Lie algebra. In this case, note that by (14) $\mathfrak {h}$ is a Frobenius algebra whose orbit through $\lambda :=\xi |_{\mathfrak {h}}$ is open, and $X_{\lambda }:=\xi +\mathfrak {h}^{\circ }$ is a Poisson transversal to the coadjoint orbit of complementary dimension. Thus the corollary implies the main result of [19].

### Proof

Note that $\{\lambda \}$ is itself a Poisson transversal, with conormal bundle $\mathfrak {h}\times \{\lambda \}$, and that the pullback of ${\varOmega }_{\mathfrak {h}}$ under $i_{\mathfrak {h}\times \{\lambda \}}:\mathfrak {h}\rightarrow \mathfrak {h}\times \{\lambda \}$, $x\mapsto (x,\lambda )$ is given by $\omega _{\lambda }$. Thus, Theorem B specializes to the diffeomorphism claimed in (a).

The conormal bundle of $X_{\lambda }$ is $N^*X_{\lambda }=\mathfrak {h}\times X_{\lambda }$, and the relevant two-form restricted to this space, $\sigma :=-{\varOmega }_{\mathfrak {g}}|_{\mathfrak {h}\times X_{\lambda }}$, is given by

$$\begin{aligned} \sigma ((x,\xi ),(y,\eta ))_{(x_0,\xi _0)}&=\eta ({\varXi }_{x_0}x)-\xi ({\varXi }_{x_0}y)-\xi _0([{\varXi }_{x_0}x,{\varXi }_{x_0}y])\\&=-\lambda ([{\varXi }_{x_0}x,{\varXi }_{x_0}y]), \end{aligned}$$

where we have used that $TX_{\lambda }=\mathfrak {h}^{\circ }\times X_{\lambda }$, that $\xi _0|_\mathfrak {h}=\lambda$ and that ${\varXi }_{x_0}(\mathfrak {h})\subset \mathfrak {h}$. Hence, $\sigma =\mathrm {pr}_1^*(\omega _{\lambda })$, and (20) becomes Theorem 4 for the Poisson map *r* and the canonical sprays. This proves (b) in a neighborhood of $\{0\}\times X_{\lambda }$, respectively, $\{0\}$. We will conclude that (b) holds on the entire $\mathcal {U}\times X_{\lambda }$ after we prove part (d).

Part (c) is a direct consequence of Theorem 6.

The stabilizer group $H_{\lambda }$ of $\lambda$ is discrete, and therefore, the map $h\mapsto h\cdot \lambda =\mathrm {Ad}_{h^{-1}}^*\lambda$ is a local diffeomorphism from *H* to *S*, inducing the diffeomorphism $H/H_{\lambda }\cong S$. Note also that by (9) $-{\varOmega }_{H}|_{H\times \lambda }=\mathrm {d}\widetilde{\lambda }$, where $\widetilde{\lambda }$ is the left-invariant one-form extending $\lambda$. Therefore, restricting the right side of (12) to $H\times \{\lambda \}$, respectively $\mathfrak {h}\times \{\lambda \}$, we obtain the following commutative diagram of local symplectomorphisms:23In particular, this shows that $\mathcal {U}\subset \mathcal {O}(\mathfrak {h})$. Also, this implies that we have an induced symplectomorphism:Since *r* is *H*-equivariant, it follows that $\mathfrak {g}^*|_S$ is *H*-invariant. Moreover, since *S* is the $\lambda$-orbit of *H*, it follows easily that the map $H\times X_{\lambda }\rightarrow \mathfrak {g}^*|_S$, $(h,\xi )\mapsto \mathrm {Ad}_{h^{-1}}^* \xi$ induces an *H*-equivariant diffeomorphism:which satisfies $r\circ {\varPsi }=\psi \circ \mathrm {pr}_1$. To prove that ${\varPsi }$ is indeed a Poisson isomorphism, note that both Poisson structures are *H*-invariant, and ${\varPsi }$ is *H*-equivariant. Therefore, it suffices to check that ${\varPsi }$ is a Poisson map in a neighborhood of $(H_{\lambda }\times X_{\lambda })/H_{\lambda }$, and this follows from the commutativity of diagram (23), and that of diagram (20) around $\{0\}\times X_{\lambda }$. On the other hand, we can now reverse the argument: having proven that (21) is a commutative diagram of Poisson maps, it follows that (20) is a Poisson map on the entire $\mathcal {U}\times X_{\lambda }$, respectively, $\mathcal {U}$, and hence (b) holds. Hence the factorization from (d) holds:and this concludes the proof. $\square$

### Proof (of Corollary 1)

By Lemma 1, each fiber $X_{\mu }:=r^{-1}(\mu )$, $\mu \in S$, is a Poisson transversal; or equivalently, $\pi _{\mathfrak {g}}$ is horizontally nondegenerate for the map (22).

For $\mu \in S$, we have that $N^*X_{\mu }=\mathfrak {h}\times X_{\mu }$. Therefore, the normal bundle is given by the tangent space to the $\mathfrak {h}$-orbits:

24 $$\begin{aligned} N_{\xi }X_{\mu }=\pi _{\mathfrak {g}}^{\sharp }(N^*_{\xi }X_{\mu })=\pi _{\mathfrak {g}}^{\sharp }(\mathfrak {h}\times \{\xi \})=\{\mathrm {ad}_x^*\xi : x\in \mathfrak {h}\}. \end{aligned}$$

Since the horizontal distribution is precisely the canonical normal bundle to the fibers, this implies (1).

Diagram (21) implies that the Poisson structure on $\mathfrak {g}^*|_S$ decomposes as a sum of two commuting Poisson structures $\pi _{\mathfrak {g}}|_{r^{-1}(S)}={\varPsi }_*(\pi _{X_{\lambda }})+ {\varPsi }_*(\mathrm {d}\widetilde{\lambda }^{-1})$, and since ${\varPsi }_*(\pi _{X_{\lambda }})$ is tangent to the fibers of *r*, and ${\varPsi }_*(\mathrm {d}\widetilde{\lambda }^{-1})$ is tangent to the *H*-orbits, it follows that this is precisely the decomposition into vertical plus horizontal bivectors:

$$\begin{aligned} \pi ^{\mathrm {v}}:={\varPsi }_*(\pi _{X_{\lambda }}), \ \ \ \pi ^{\mathrm {h}}:={\varPsi }_*(\mathrm {d}\widetilde{\lambda }^{-1}), \end{aligned}$$

which proves (3). Since *r* is a Poisson map, it follows that $\pi ^{\mathrm {h}}$ projects to $\pi _{\mathfrak {h}}$, and therefore, the inverse of $\pi _{\mathfrak {h}}|_S$ (i.e., the symplectic structure on *S*) pulls back to the inverse of $\pi ^{\mathrm {h}}$ restricted to annihilator of the fibers (i.e., the horizontal two-form). This implies (2) (see also [20, Proposition 3.6]). $\square$

### Proof (of Corollary 2)

By (24) it follows that the horizontal lift of the corresponding Ehresmann connection is given by:

$$\begin{aligned} \mathrm {hor}_{\xi }:T_{\mu }S\longrightarrow N_{\xi }X_{\mu }, \ \ \mathrm {hor}_{\xi }(\mathrm {ad}_x^*\mu )=\mathrm {ad}_x^*\xi , \ \ \xi \in X_{\mu }, \end{aligned}$$

and so

$$\begin{aligned} \pi _{X_{\mu },\xi }=\pi _{\mathfrak {g},\xi }-\pi ^{\mathrm {h}}_{\xi }=\pi _{\mathfrak {g},\xi }-(\wedge ^2\mathrm {hor}_{\xi })(\pi _{\mathfrak {h},\mu }), \ \ \xi \in X_{\mu }. \end{aligned}$$

The claim now follows from the fact that the horizontal lift has an affine dependence on $\xi \in X_{\mu }$. $\square$

## Footnotes

1 Following a suggestion of the referee, let us also point out, as a guide for the reader to Theorems 2 and 3, that in symplectic geometry one has tubular neighborhood theorems for symplectic and Lagrangian submanifolds. Now Lie-Dirac submanifolds (of which Poisson transvsersals are examples) correspond to symplectic subgroupoids [4, Theorem 9], while the graph of a Poisson map $\varphi : (M_0,\pi _0) \rightarrow (M_1,\pi _1)$, sitting as a coisotropic submanifold in $(M_0,\pi _0) \times (M_1,-\pi _1)$, corresponds to a Lagrangian subgroupoid [2, Theorem 5.4].

2 Note that our sign convention is different from that in [1]; namely, the IM-form corresponding to a closed two-form $\eta$ on a groupoid $\mathcal {G}$, is given by $A\ni V\mapsto \mathbf {u}^*(-\iota _V\eta )$, where $\mathbf {u}:M\rightarrow \mathcal {G}$ is the unit map.
